# Continuing Cancer Therapy through the Pandemic While Protecting Our Patients: Results of the Implementation of Preventive Strategies in a Referral Oncology Unit

**DOI:** 10.3390/cancers13040763

**Published:** 2021-02-12

**Authors:** Michalis Liontos, Efstathios Kastritis, Christos Markellos, Magdalini Migkou, Evangelos Eleftherakis-Papaiakovou, Konstantinos Koutsoukos, Maria Gavriatopoulou, Flora Zagouri, Theodora Psaltopoulou, Evangelos Terpos, Meletios-Athanasios Dimopoulos

**Affiliations:** Department of Clinical Therapeutics, National and Kapodistrian University of Athens, Alexandra Hospital, Athens 11528, Greece; ekastritis@med.uoa.gr (E.K.); chrismarkellos@hotmail.com (C.M.); mmigkou@med.uoa.gr (M.M.); evelepapa@med.uoa.gr (E.E.-P.); konkoutsoukos@med.uoa.gr (K.K.); mgavria@med.uoa.gr (M.G.); fzagouri@med.uoa.gr (F.Z.); tpsaltop@med.uoa.gr (T.P.); eterpos@med.uoa.gr (E.T.); mdimop@med.uoa.gr (M.-A.D.)

**Keywords:** cancer, COVID-19, SARS-CoV-2, screening

## Abstract

**Simple Summary:**

Cancer patients are vulnerable to the SARS-CoV-2 infection. Their treatment has also been negatively affected by the COVID-19 pandemic. No solid data exist regarding the appropriate management of cancer patients during the pandemic. Our center, a referral oncology/hematology unit, has implemented specific preventive and screening measures. This study aimed to record the epidemiological characteristics of our patients with cancer that were detected positive for SARS-CoV-2 by molecular testing. Since June 2020, 11,618 patient visits were performed in our unit, and only 26 patients were detected positive for SARS-CoV-2, corresponding to a 0.22% positivity ratio. Among asymptomatic patients committed to begin a new line of systemic therapy, only four were found positive. No transmission within the unit was found after detailed tracing of positive patients. These data will help to update guidelines and recommendations in order to improve cancer care during the current pandemic.

**Abstract:**

Cancer patients infected with SARS-CoV-2 have worse outcomes, including higher morbidity and mortality than the general population. Protecting this vulnerable group of patients from COVID-19 is of the utmost importance for the continuous operation of an oncology unit. Preventive strategies have been proposed by various societies, and centers around the world have implemented these or modified measures; however, the efficacy of these measures has not been evaluated. In our center, a referral oncology/hematology unit in Athens, Greece, we implemented strict protective measures from the outset of the pandemic in the country and we have prospectively recorded the epidemiological characteristics of COVID-19. Among 11,618 patient visits performed in our unit, 26 patients (case-to-visit ratio of 0.22%) were found positive for SARS-CoV-2, including 4 (1%) among 392 patients that were screened before starting primary systemic treatment. Among patients tested positive for SARS-CoV-2, 22 were symptomatic at the time of diagnosis; subsequently, 12 required hospitalization and 5 died due to COVID-19. Detailed contact tracing indicated that there was no in-unit transmission of the infection. Thus, strict implementation of multilevel protective strategies along with a modestly intense screening program allowed us to continue cancer care in our unit through the pandemic.

## 1. Introduction

The COVID-19 pandemic has spread to almost every country within less than a year, infected millions of people, led to more that 1.5 million deaths so far [1], and substantially affected every aspect of everyday life activities. In the Western Hemisphere, two distinct waves of the pandemic can now be clearly seen. The first, during spring 2020, caught governments and health care providers by surprise, leading to dramatic effects in certain countries or regions regarding the number of patients admitted in intensive care units (ICUs) and deaths [2]. In other countries, including Greece, the number of infections and deaths remained low, but strict quarantine measures were implemented. The second wave of the pandemic affected more countries, and despite prior experience, the number of infections rose substantially, associated also with a significant rise in infection-related deaths. As expected in a pandemic, health care has been the most heavily affected sector. Not only have health care workers paid a heavy death toll working in close proximity to infected patients [3], but they have also had to completely transform their activities in order to ensure continuous care for the remaining groups of patients [4]. We have already shown that the latter has significantly decreased the number of visits in health care facilities among patients with chronic illnesses to receive treatment and appropriate follow-up, or to manage disease or treatment complications [5].

It is conceivable that the above has affected all aspects of cancer treatment (systemic therapies, surgery, radiotherapy, rehabilitation, etc.) [6,7], as well as screening programs [8]. However, cancer patients face additional challenges: they are usually of more advanced age than the general population, and their underlying disease and treatment may be associated with an increased risk of acquiring the infection and developing more severe complications; the frequent visits in health care facilities further increase their risk of exposure to the virus [9]. The optimal strategy to manage treatment needs, follow-up, and cancer screening, and to protect cancer patients from the infection, has not yet been defined. However, most cancer centers have developed strategies to mitigate risks and continue patient care. Our center, a tertiary referral unit, has developed and implemented such strategies since the beginning of the pandemic, and has updated them accordingly as the characteristics of the pandemic change and testing becomes more widely available [10]. 

In this report, we present the results from the epidemiological surveillance in our unit during the two phases of the pandemic, in order to provide additional data that could help optimize strategies for the care of cancer patients and their health care providers. 

## 2. Materials and Methods

### 2.1. Study Population

The study included patients that are followed and treated at the Oncology/Hematology Unit of the Department of Clinical Therapeutics. Our unit is a tertiary referral center for plasma cell dyscrasias (especially myeloma, Waldenström Macroglobulinemia, and amyloidosis), and genitourinary and gynecological malignancy treatment. We follow several thousands of patients each year. 

In the current analysis, we included all patients who had visited the Oncology Unit since 1 June 2020 in order either to receive intravenous systemic treatment (chemotherapy, targeted agents, immunotherapy) or to be prescribed orally administered medication. In addition, patients that were on follow-up for their disease at that period of time were included in the analysis, irrespective of their last visit to the unit. Surveillance data for COVID-19 infection were collected prospectively; the demographic and main disease (cancer) characteristics were collected by review of the medical records of the patients. 

For all positive cases, including health care workers and patients, the tracing of close contacts was performed by the Infections Committee of our hospital, following local and national guidelines. Central follow-up of close contacts by the relevant national authorities was also implemented, which is a standard approach in the country. All persons considered close contacts of a positive case were quarantined for 14 days and tested for SARS-CoV-2 in case of symptoms appearance. Standard descriptive statistics were used to summarize clinical-, disease-, and treatment-related characteristics of the patients. 

### 2.2. Description of the Preventive Measures

Following guidance from the Greek Health Care Authorities, international organizations, and oncological societies, a series of protective measures were implemented by the Oncology/Hematology Unit and were approved by the Infection Committee of our hospital. These measures have already been published along with their results up to June 2020 [10], and are described in more detail in Supplemental Table S1. In brief, the following actions were implemented: all patients receiving intravenous chemotherapy had to practice appropriate hand hygiene and wear masks for the entire time that they remained in the hospital premises. For this purpose, alcohol-based sanitizers were made available in every room of the Oncology Department (including chemo rooms and corridors, waiting areas, etc.) and in all public areas. A dedicated person followed and ensured that patients and their aides practiced these measures at least within the unit. Physical distancing practices were exercised in the waiting room area, including frequent inspections to avoid crowding. During chemotherapy administration, patients were positioned in chairs maintaining a safe distance from all other persons whenever possible. Only patients scheduled to receive treatment were allowed in the waiting area. All visitors and patient aides were instructed to remain outside the facility. Signs clearly listing COVID-19 symptoms were provided at all entrances of the Oncology Department. Signs also instructed patients/visitors with any of these symptoms not to enter the premises and follow the designated pathway for further evaluation. Alternative shift schedules among physicians, oncology nurses, and study coordinators were followed in order to limit each individual’s exposure and increase distancing. This also allowed the extension of unit’s working hours in order to accommodate all patients during a more extensive timeframe, thus further avoiding congestion. Finally, digital solutions were sought for the academic activities and clinical trials conducted in the unit. More specifically, didactic academic teaching and departmental meetings were conducted using video conferencing. Additionally, regular site monitoring visits were carefully scheduled in order to avoid crowding and ensure distancing between monitors. Monitoring activities were also conducted remotely by digital means that replaced in-person interaction.

### 2.3. Screening Program

In order to detect asymptomatic and pre-symptomatic COVID-19 cases, our unit implemented screening guidelines that were approved by the Ethics Committee of our hospital. More specifically, all health care professionals, including physicians, nurses, and administration personnel who reported no symptoms associated with COVID-19 or any contact history with a confirmed or suspected case, were molecularly tested for COVID-19 every 7 days. In addition, all patients committed to begin a new line of treatment were tested for COVID-19 prior to the first cycle of therapy. Patients with symptoms were not allowed to enter the unit, and were evaluated in a separate dedicated room, where appropriate molecular testing was performed. Since October 2020, a rapid antigen test was added to the initial evaluation. 

## 3. Results

### 3.1. Study Population

For the purposes of the current report, the data cut-off day was 10 December 2020; since 1 June 2020, 11,618 patient visits were performed in our unit, corresponding to approximately 85 patient visits per day. The vast majority of the visits were performed for administration of parenteral systemic therapy, while approximately 20% of the visits were performed for disease re-evaluation and/or refilling of prescriptions for oral medications. During the same period, 392 patients who started a new line of therapy were screened for SARS-CoV-2 infection with a molecular test (PCR) prior to treatment initiation. Twenty-two patients among those receiving treatment or being followed-up in the unit tested positive after developing COVID-19-related symptoms. Thus, the case-to-visit ratio was 2.2 cases per 1000 visits (0.22%). Four patients (1.0%) tested positive for SARS-CoV-2 through this screening process; all were asymptomatic. During the same period, 6/64 (9.4%) health care professionals tested positive at regular screening (all were asymptomatic). During the same period, 118,335 cases of COVID-19 were diagnosed in Greece, corresponding to a rate of approximately 1.18 cases per 1000 population [11]. 

Patient characteristics, treatments, and the outcomes of infected patients are presented in Table 1. Briefly, the male-to-female ratio was 1:1 and the median age was 59.5 years (range 48–81 years). Most of the infected patients, (14/26; 53.8%) had plasma cell dyscrasias (11 had myeloma, 2 had Waldenström’s macroglobulinemia, and one had amyloidosis). Ovarian cancer was the most common neoplasm among the remaining patients (N = 4, 15.4%). This distribution of underlying malignancies is in line with the patient population that is referred to our unit, with the majority being treated and followed for plasma cell dyscrasias.

Hospitalization for COVID-19 was required in 12/26 (46.2%) patients. Among those that required hospitalization, six had plasma cell dyscrasias (five had myeloma and one, Waldenström’s macroglobulinemia), while the remaining six patients had rectal, lung, ovarian, and vulvar cancer, non-Hodgkin lymphoma, and cancer of unknown primary, respectively; 18/26 (69.2%) patients had metastatic or recurrent disease, and these accounted for the majority of patients that were hospitalized (9/12 patients, 75.0%). Patients that did not require hospitalization received no specific treatment. Hospitalized patients received a variety of specific anti-COVID treatments in accordance with the treatment protocols and clinical trials available at the time of hospitalization at each site (Supplemental Table S2). Finally, 5/22 (22.7%) symptomatic patients died (overall 5/26, 19.3% of those diagnosed with the infection); most patients that died (4/5 patients, 80.0%) had recurrent or metastatic disease; only one patient had myeloma. A minority of patients (6/26 patients, 23.1%) had impaired performance status at COVID-19 diagnosis (ECOG-PS ≥ 2), but five out of six were hospitalized, and four of them died due to SARS-CoV-2 infection. All patients that died were symptomatic at the time of initial testing. The four patients detected through the screening program were asymptomatic and none developed any COVID-19-related symptoms during follow-up. 

At data cut-off, COVID-19 had resolved in 20 patients and disease was ongoing for 1 patient. Among infected patients, 13 were receiving active treatment at the time of infection and 9 of them (69.2%) resumed treatment after resolution of symptoms, or, for the four asymptomatic patients, completion of at least 14 days in quarantine. 

### 3.2. Contact Tracing 

COVID-19 transmission within an oncology unit could have devastating consequences, and every effort must be taken to mitigate this risk. To evaluate possible transmission of SARS-CoV-2 within the oncology unit, despite the preventive measures that were implemented, and also to identify potential weakness in the preventive strategy, we meticulously traced all suspected contacts in each of the positive cases. For the four cases that tested positive at screening and thus did not enter the unit, no transmission within the unit occurred. Among the remaining 22 cases, the median time from last visit to COVID-19 symptoms and testing was 20 days (interquartile range, 51 days); however, only 4 out of the 22 patients tested positive within 10 days of their last visit to the unit (Table 2). Detailed and meticulous tracing of patients that tested positive for SARS-CoV-2 indicated that there was no in-unit transmission for any of them, and that suspected contacts were outside the unit in all cases; in four cases, a close contact was already known to be positive for SARS-CoV-2 at the time of testing. None of the personnel that tested positive contacted the virus in the unit, but instead during their duties in the ER or other wards of the hospital. Additionally, there were no confirmed or suspicious cases of transmission from personnel to patients or from oncology unit patients to personnel. 

## 4. Discussion 

Cancer patients have been severely affected by the COVID-19 pandemic in at least two ways: they are at increased risk for acquiring the infection and developing severe disease, but also, the care for their underlying malignant disease may be affected by the re-allocation of resources and difficulties in reaching the hospital and specialized care facilities. While increases in resources both to cover the needs of pandemic management and to retain the current status of health care may not be feasible, strategies to mitigate the risk of cancer patients acquiring the infection are realistic and can be immediately effective. The re-allocation of heath care system resources in order to cope with the increasing number of COVID-19 patients has substantially affected screening, diagnostic, and treatment options for patients with chronic illnesses, especially cancer. Additionally, cancer patients—especially those under active treatment—have higher morbidity and mortality from COVID-19 [9]. Therefore, continuing operation of oncology units during the pandemic and preventing transmission of SARS-CoV-2 among health care professionals and patients are of primary importance.

The data presented here describe the experience of our oncology unit, which continued operations uninterrupted during the pandemic. Although the initial pandemic wave did not severely affect Greece, the second wave was substantially harder. However, the early implementation of the preventive measures in our unit resulted in a low rate of infection even in the second wave, which started in September. These measures included strict protective strategies both for personnel and patients early in the course of the pandemic [10] that were further refined, evolved, and updated with the developing situation and the publication of international guidelines. 

Ideally, health care facilities have two lines of defense against SARS-CoV-2 transmission. The first is continuous testing for SARS-CoV-2 to identify asymptomatic or symptomatic patients among personnel, patients, and visitors; the second is the implementation of protective measures to prohibit transmission. Scientific societies and health policy makers have emphasized the value of screening cancer patients under active treatment [7,12], but screening intensity is a matter of debate, taking into consideration available resources and potential delays. A recent study evaluated the role of performing sequential molecular testing for SARS-CoV-2 in both cancer patients and health care professionals [13]. Cancer patients under intravenous therapies were tested prior to each cycle of therapy, and site staff were tested at regular intervals. The results of the study were alarming, since 30% of patients and 25% of the unit’s staff were infected. Although most had mild symptoms, 12.5% of the patients died of COVID-19. Other universal screening approaches have also resulted in a significant percentage of COVID-19 positive patients. More specifically, a screening approach in the United Arab Emirates with RT-PCR testing in all cancer patients resulted in a 7.5% positivity rate [14]. Analogously, a two-step screening approach consisting of serological screening followed by RT-PCR test for positive cases resulted in almost 10% positive cases among cancer patients in Bergamo during the first wave of the pandemic [15]. In our study, a non-intensive approach was followed, and molecular testing was offered as a screening method only to cancer patients committed to begin a new treatment. Following this approach, the percentage of positive patients was significantly lower (1.0%) and was in accordance with previous reports from the first wave of the pandemic [16,17,18,19]. There are several factors that may contribute to the small number of patients that were detected positive for SARS-CoV-2 in our study. First, the spread of the infection in the community was less than in other countries; second, screening frequency was less intensive; and third, patients diagnosed with neoplastic diseases tend to be more cautious and follow hygiene and social distancing measures to a greater extent than the general population. However, screening identified a few cases; all were asymptomatic. 

The protective measures followed in our unit were in compliance with issued guidelines [20,21]. The significance of social distancing, hygiene measures, and workload optimization have been shown [22,23,24]. No surge of positive cases—indicative of transmission within the unit—was detected from our epidemiological data. This was the case even for the very few patients that developed symptoms and tested positive for SARS-CoV-2 a few days after their last visit to the unit. This highlights that the implementation of strict protective measures, with a modest-intensity screening program, can allow for the safe administration of optimal cancer care [18,23]. It should be noted that the design of this study, as well as the government-established tracing methodology, did not allow for asymptomatic transmission detection. However, it is thought that the presence of asymptomatic transmission would soon result in an outbreak of cases among treated patients, since they are a vulnerable, high-risk population. No such surge of cases was confirmed in our unit. 

Despite the small number of patients diagnosed with COVID-19, the outcome indicates a significant risk for cancer patients of developing complications and dying, in line with other studies that have indicated their vulnerability to COVID-19 [25,26,27] and possible adverse prognostic factors [25,28]. Patients in impaired clinical condition at the time of infection had worse outcomes, in accordance with previous data [29]. Most patients with SARS-CoV-2 infection at our site, and half of the symptomatic patients requiring hospitalization, had been diagnosed with plasma cell dyscrasias, although they accounted for only one of five recorded deaths. Our unit has a special interest and is a referral center for these diseases, explaining the overrepresentation of these patients among those infected with SARS-CoV-2. Patients with plasma cell dyscrasia, such as myeloma, are usually severely immunocompromised, and thus at risk for severe COVID-19 infection, as recently shown [30,31,32,33]. Some recent data indicate that optimal myeloma management may confer protection against an adverse outcome with COVID-19 [34]. However, due to the small number of cases, we cannot draw firm conclusions about the severity and outcome of COVID-19 in patients with plasma cell dyscrasias. 

Treatment resumption post-COVID-19 is an important issue for cancer patients. All asymptomatic patients diagnosed with the disease during screening, as well as most of the symptomatic patients that recovered from the infection, resumed treatment without sequalae. This adds to previously published data [35], and enhances the notion that cancer patients may safely continue their treatment post-COVID-19, following thorough evaluation by their treating physician, and after at least a 14-day period from symptomatic resolution. Recent publications and regulatory approvals of SARS-CoV-2 vaccines [36,37] have spread optimism that this pandemic could eventually be controlled. However, very limited data exist regarding immunocompromised patients. Therefore, longitudinal screening and serological studies, multicenter collaboration, and data sharing are required to better understand the special needs of and risks to cancer patients, the role of anticancer therapies, and COVID-19.

## 5. Conclusions

A combination of strict protective measures and a modest-intensity screening program for SARS-CoV-2 allowed the safe and uninterrupted operation of our oncology unit during the pandemic, with a low case-to-visit ratio. This data, in combination with the experience gathered worldwide, will enable the optimization of preventive strategies and help to update guidelines and recommendations from the oncology hematology societies to improve cancer care during the current pandemic. Despite the optimism for the future that is associated with the recent approval of vaccines and the initiation of vaccination programs, our cancer patients may still need to be managed within a “safety net” for several more months. 

## Figures and Tables

**Table 1:** Clinical characteristics of the patients.

Patient Number	Age	Sex	Type of Cancer	Recurrent/Metastatic Disease	Symptomatic	Smoking	Comorbidities	Hospitalization	ICU admission	ECOG-PS	Ongoing Antineoplastic Treatment	Outpatient Visits Since June 2020	Survival Status
1	74	M	Rectal cancer	No	Yes	Yes	Renal insufficiency, COPD, Diabetes mellitus, dementia	Yes	No	2	None	0	Dead
2	70	F	Ovarian cancer	Yes	Yes	No	Atrial fibrillation	Yes	No	2	Paclitaxel	12	Alive
3	64	M	Waldenstrom macrglobulinemia	Yes	Yes	Yes	Arterial Hypertension, depression	Yes	Yes	0	None	3	Alive
4	68	M	Multiple Myeloma	Yes	Yes	NA	Coronary artery disease	Yes	Yes	2	Bortezomib-Cyclophosphamide-Dexamethasone	16	Dead
5	60	F	Multiple Myeloma	Yes	Yes	Yes	Obesity	No	NA	0	Daratumumab-lenalidomide-dexamethasone	8	Alive
6	80	M	Multiple Myeloma	Yes	Yes	No	Arterial hypertension, dyslipidemia	Yes	No	0	None	6	Alive
7	81	F	Multiple Myeloma	Yes	Yes	No	COPD, Arterial Hypretension	No	NA	3	Ixazomib-cyclphosphamide-dexamethasone	3	Alive
8	67	M	Multiple Myeloma	Yes	Yes	No	None	No	NA	0	Carfilzomib-lenalidomide-dexamethasone	15	Alive
9	49	F	Multiple Myeloma	Yes	Yes	NA	Obesity	Yes	No	0	Ixazomib-cyclphosphamide-dexamethasone	8	Alive
10	57	F	Vulvar cancer	No	Yes	No	Hepatitis B, Obesity	Yes	No	1	Cisplatin as part of CCRT	6	Alive
11	75	M	Lung cancer	Yes	Yes	Yes	Obesity	Yes	Yes	0	Gemcitabine-Csiplatin-Pembrolizumab	6	Dead
12	53	F	Cancer of Unknown Primary	Yes	Yes	Yes	None	Yes	No	3	Capecitabine	6	Dead
13	48	F	Breast Cancer	No	No	No	None	No	NA	0	None	0	Alive
14	52	F	Waldenstrom macrglobulinemia	No	Yes	No	Arterial hypertension, dyslipidemia, obesity	No	NA	0	None	2	Alive
15	72	F	Non-Hodgkin Lymphoma	Yes	Yes	No	Arterial hypertension, dyslipidemia, diabetes mellitus	Yes	No	2	Rituximab-lenalidomide	8	Dead
16	57	M	Multiple Myeloma	No	Yes	No	Arterial hypertension, dyslipidemia, hypotheroidism	No	NA	0	Bortezomib-thalidomide-dexamethasone	5	Alive
17	59	M	Multiple Myeloma	No	Yes	No	Non-alcoholic fatty liver disease	No	NA	0	None	0	Alive
18	57	M	Prostate cancer	Yes	Yes	Yes	None	No	NA	0	Leuprolide	0	Alive
19	55	M	Ovarian Cancer	Yes	No	No	Obesity	No	NA	0	None	0	Alive
20	51	F	Ovarian cancer	Yes	Yes	Yes	None	No	NA	0	Letrozole	4	Alive
21	58	M	Multiple Myeloma	No	Yes	No	Arterial hypertension	Yes	No	0	Lenalidomide-Zoledronic acid	3	Alive
22	48	M	Renal cancer	Yes	No	Yes	Arterial hypertension	No	NA	0	None	0	Alive
23	64	M	Multiple Myeloma	No	No	Yes	None	No	NA	0	Lenalidomide	2	Alive
24	65	F	Multiple Myeloma	Yes	Yes	No	Arterial Hypertension, Thyroidectomy, arrhythmia	Yes	No	1	Daratumumab-lenalidomide-dexamethasone	11	Alive
25	54	F	Ovarian Cancer	No	Yes	No	None	No	NA	1	Paclitaxel-Carboplatin	3	Alive
26	64	F	Amyloidosis	Yes	Yes	No	Hypothyroidism, Dyslipidemia, Chronic Renal Failure	No	NA	1	Pomalidomide-Cyclophosphamide-Dexamethasone	0	Alive

**Table 2:** Tracing of cases.

Patient Number	Age	Sex	Known Close Contact Tested Positive	Interval from Last Outpatient Visit (Days)
1	74	M	0	365
2	70	F	0	2
3	64	M	0	97
4	68	M	0	14
5	60	F	0	13
6	80	M	0	26
7	81	F	0	60
8	67	M	0	14
9	49	F	0	8
10	57	F	0	20
11	75	M	0	5
12	53	F	1	21
13	48	F	0	Diagnosed at screening
14	52	F	0	26
15	72	F	0	6
16	57	M	1	11
17	59	M	1	270
18	57	M	1	Diagnosed at screening
19	55	M	0	Diagnosed at screening
20	51	F	0	30
21	58	M	0	20
22	48	M	0	Diagnosed at screening
23	64	M	0	67
24	65	F	0	18
25	54	F	1	10
26	64	F	0	270

## Data Availability

The data presented in this study are available on request from the corresponding author.

## Supplementary Materials

The following are available online at https://www.mdpi.com/2072-6694/13/4/763/s1, Table S1: Protective measures implemented in the Oncology Unit., Table S2: Specific anti-COVID treatment administered among hospitalized patients.
